# Transdiagnostic Effectiveness of Repetitive Transcranial Magnetic Stimulation for Mood and Anxiety Disorders

**DOI:** 10.1192/j.eurpsy.2025.557

**Published:** 2025-08-26

**Authors:** A. V. Samokhvalov, A. Doomra

**Affiliations:** 1 Homewood Health Centre, Guelph; 2 Department of Psychiatry and Behavioural Neurosciences, McMaster University, Hamilton; 3 Dr.Sam’s Health, Inc.; 4 Homewood Research Institute, Guelph, Canada

## Abstract

**Introduction:**

Repetitive Transcranial Magnetic Stimulation (rTMS) is a novel neuromodulation treatment investigated for multiple psychiatric conditions and approved primarily for treatment-resistant depression (TRD) [1, 2]. There is a perceived potential for other clinical conditions, primarily other mood and anxiety disorders [2]. We have been using rTMS for treatment of patients with TRD of mixed etiology and multiple comorbidities.

**Objectives:**

To evaluate the effectiveness and feasibility of rTMS in complex clinical populations.

**Methods:**

Observational study. Quick Inventory of Depressive Symptomatology (QIDS). Generalized Anxiety Disorder Questionnaire (GAD-7). Descriptive statistics.

**Results:**

We have treated 90 patients, 46 women (51.1%) and 44 men (48.9%), with average age of 42.5±16.9 years. Vast majority (88.9%) of patients had a primary diagnosis of major depressive disorder, 8.9% had bipolar depression, and two patients had primary anxiety disorders. The standard questionnaires were used to quantify the severity of depressive symptoms (QIDS) and anxiety (GAD-7). The average baseline scores for depression and anxiety were 16.6±4.9 and 12.7±5.5, respectively. The patients received an average of 24.0±7.0 treatments. Almost all patients received the full course of 20-30 treatments as planned. The average end-of-treatment (EoT) scores for severity of depressive symptoms and anxiety were 10.5±6.1 and 8.4±5.8, respectively. The rates of improvement and complete resolution of depressive symptoms were 64.4% and 28.7%, respectively. The rates of improvement and complete resolution of anxiety symptoms were 53.5% and 29.6%, respectively. There was a significant difference between the bipolar and major depression in terms of baseline depressive symptoms severity and improvement rates, but there was no difference in respect to anxiety symptoms (see Figure 1).

**Image 1:**

**Figure:**
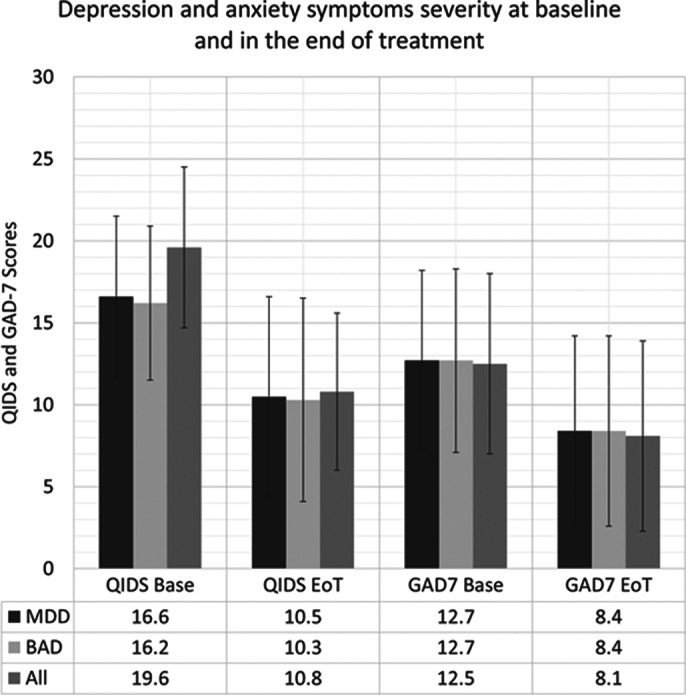

**Conclusions:**

rTMS provides significant improvement and recovery rates in complex clinical populations and is well-tolerated. We recommend wider implementation of rTMS for treatment of mood and anxiety disorders.

**References:**

1. Samokhvalov AV, Weber M. Early Outcomes of Repetitive Transcranial Magnetic Stimulation in Complex Clinical Population. Eur Psychiatry. 2023 Jul 19;66(Suppl 1):S158–9. doi: 10.1192/j.eurpsy.2023.389. PMCID: PMC10596575.
2. Milev RV, Giacobbe P, Kennedy SH, Blumberger DM, Daskalakis ZJ, Downar J, Modirrousta M, Patry S, Vila-Rodriguez F, Lam RW, MacQueen GM, Parikh SV, Ravindran AV; CANMAT Depression Work Group. Canadian Network for Mood and Anxiety Treatments (CANMAT) 2016 Clinical Guidelines for the Management of Adults with Major Depressive Disorder: Section 4. Neurostimulation Treatments. Can J Psychiatry. 2016 Sep;61(9):561-75. doi: 10.1177/0706743716660033. Epub 2016 Aug 2. PMID: 27486154; PMCID: PMC4994792.

**Disclosure of Interest:**

None Declared

